# Supplementary material for: Enhanced UV-B Radiation in Potato Stems and Leaves Promotes the Accumulation of Anthocyanins in Tubers
Source: Curr Issues Mol Biol. 2023 Dec 11;45(12):9943–60. doi: 10.3390/cimb45120621 (PMC10742819; doi:10.3390/cimb45120621)
Supplement: Supplementary file 1 [file cimb-45-00621-s001.zip › supplementary figure S1-S3.pdf]

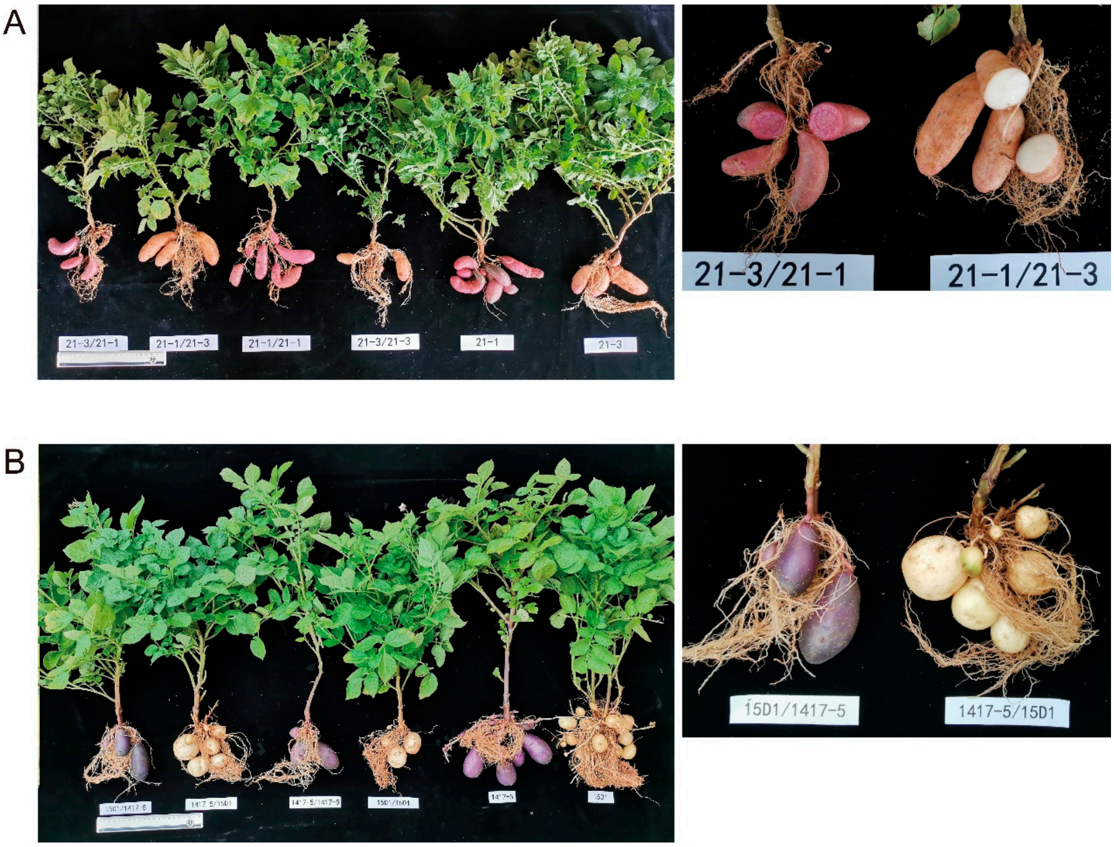

Figure S1. The photographs of the reciprocal grafting combinations with colored and uncolored potatoes. **(A)** The right shows reciprocal grafting plants with red-fleshed potato '21-1' and white-fleshed potato '21-3', and the left shows tuber color phenotype. **(B)** The right shows reciprocal grafting plants with purple-fleshed potato '1417-5' and white-fleshed potato '15D1', and the left shows tuber color phenotype.

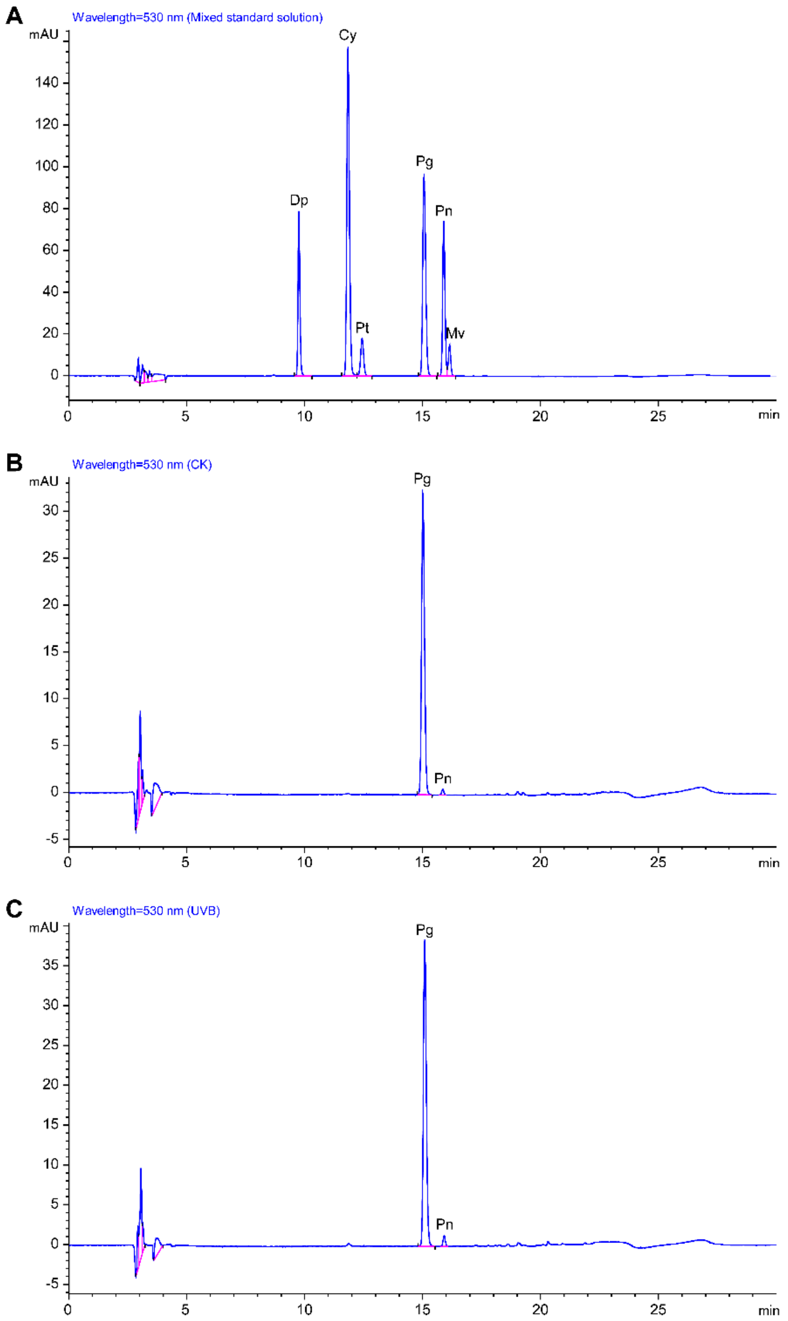

Figure S2. A representative mixed standard solution chromatogram and two tuber flesh sample solutions chromatograms. **(A)** The chromatogram of 5 mg/L mixed standard solution shows the separation of six anthocyanidins standards. **(B)** and **(C)** shows the chromatograms of CK and UVB samples, respectively. Pg and Pn were detected in CK and UVB samples.

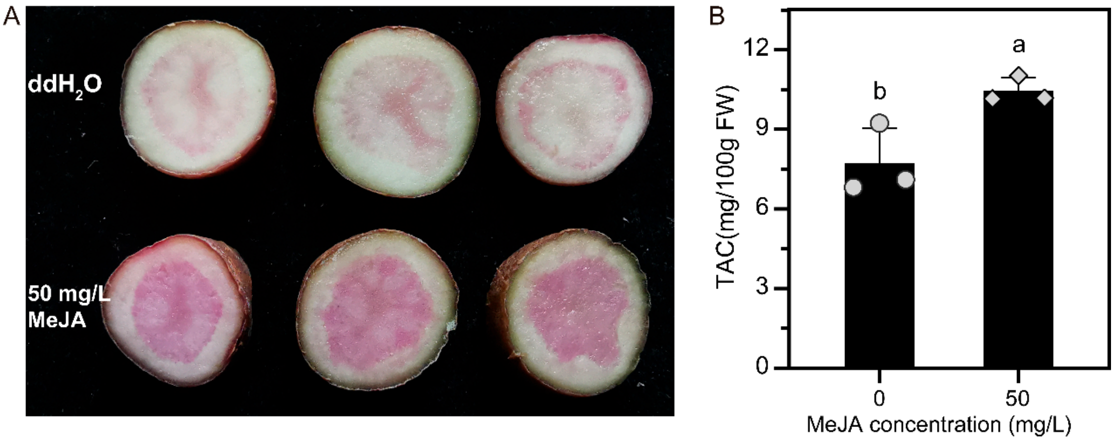

Figure S3. Exogenous methyl jasmonate (MeJA) spraying treatment. **(A)** The tuber photographs of red-fleshed potato clone '21-1' after 20 days of exogenous 50 mg/L MeJA and purified water (CK) spraying leaf. **(B)** Total anthocyanins content (TAC) of red-fleshed potato clone '21-1' after 20 days of exogenous 50 mg/L MeJA and purified water (CK) spraying leaf. The bars represent means ± SD, and the lowercase letters represent significant differences (*t*-test, n=3, p<0.05).
